# Book Reviews

**Published:** 1815-03

**Authors:** 


### CRITICAL ANALYSIS

### OF RECENT PUBLICATIONS

IN THE

### DIFFERENT BRANCHES OF PHYSIC, SURGERY, AND MEDICAL PHILOSOPHY.

---

### *Edinburgh Medical and Surgical Journal*, No. XLI. for January 1815.

OUR wish being to preserve a faithful register of every transaction in the healing art, we return now to that kind of notice of the Edinburgh Journal with which we commenced on its first changing its form from the Annual Commentaries to a trimestral publication. By reserving the whole for our review department, we are so enabled to condense its contents as to find room for that quantity of matter which now flows in upon us from all quarters, and obliges us to economise room in order to complete our promise of an Universal Medical Gazette.

The first article is a paper, by Professor Colles, on the Operation of tying the Subclavian Artery. This we should have perused with much more satisfaction, had the operation succeeded in a single instance in which it was tried. We mean not to condemn the attempt; on the contrary, we conceive nothing more laudable than a bold undertaking in a case which must prove fatal without speedy relief. We shall not offer any epitome of the operation, or the subsequent symptoms, as these will be carefully examined by any surgeon who may hereafter make the same hazardous attempt. The first patient, for whom it was tried on account of aneurism in the right subclavian artery, lived to the ninth day after the operation, and the following are all the appearances which could be ascertained after death.

"In a few hours after his death, we hastily removed the heart and large vessels, so low down as the clavicle, being prevented, by the attendance of his friends, from examining the parts *in situ*. No blood was effused into either cavity of the thorax. The lungs of both sides were sound; the upper extremity of the right lung had a slight adhesion with the parts in the vicinity of the ligature. The aorta was slit up from the heart to the forking of the arteria inominata; and thus we had an opportunity of observing the very diseased condition of the coats of this artery, as manifested by the wrinkled state of its lining membrane. A very remarkable pouch or recess, capable of receiving the point of the little finger, appeared peared in the wall of the aorta, on the right side of the artery, about one inch above the heart.

"In the apex of this dilatation, the coats of the artery were extremely thin, as if the middle coat, having been removed by absorption, the lining and outer coats had adhered together. The arteria inominata was dilated at the place of its forking. Between this dilatation and the aneurism for which the operation had been performed, there was not above a quarter of an inch of the subclavian artery free from disease, and on this part the ligature had been applied. A pretty large opening had been made by ulceration, through the coats of the artery, immediately above the ligature."

The examination of the next case is not less worthy of notice, as the subject died without undergoing the operation.

"The next case of disease requiring the operation of tying the subclavian artery, occurred in William Levanee, an invalided soldier, who was affected with aneurism of the axillary artery a little below the clavicle. The disease seemed to have arisen spontaneously. Tumour was the size of a pullet's egg at the time of his applying for relief.

"In full consultation, the operation was decided on; but this man refused to submit to it, unless we assured him of complete success. He lingered for some months, and appeared to die, not from hæmorrhage, but rather from extensive suppuration, and long-continued irritation.

"*Appearances on dissecting the Body of William Levanee, ætat. annos* 55.—26th April, 1813. Very considerable swelling occupied the right side of the body, extending from the clavicle down to the eighth rib, and from the nipple of the breast across the arm. The arm and fore-arm were very much distended by an œdematous swelling. Three large ulcers a little below and before the axilla; inferior costa of the scapula rough on its internal surface; the inferior angle of this bone exposed and rough. On opening the thorax, the viscera were found perfectly healthy, with the exception of slight adhesions of the right lung to the pleura costalis, and some water in that side of the chest. Heart rather small, but appeared perfectly healthy. Large arteries near the heart not at all dilated, and no other traces of disease in any of them than some very small opake spots visible on their internal coats.

"The pectoralis major was of a pale brown colour, and much thinner than natural; the pectoralis minor converted into a substance resembling a strong fascia. The parts above the clavicle were still in a healthy state; the different steps of the operation for tying the subclavian artery before it had reached the scaleni were performed on it. The trunk of this artery was traced of natural size and healthy texture, for an inch and a half below the origin of the vertebrals, and running over the front of the aneurismal tumour. At this place, its coats became so thin, that it was no longer possible to distinguish them from the coats of the tumour. Immediately

Immediately before it became indistinct, this part of the artery seemed dilated to the breadth of a quarter of an inch. The articulation of the humerus was opened, and communicated with the aneurismal tumour. The branches of the subclavian artery did not appear at all enlarged. The basilic vein was thickened in its coats, but not at all enlarged in its diameter."

Of the next case the particulars could be collected with more accuracy, as the subject was a clergyman, and in that rank of life in which every ailment is early attended to. As far as we can learn, the first symptom was numbness in the right arm, enlargement of the hand, or a sensation like it, and afterwards pain about the middle of the fore arm, or insertion of the deltoid muscle. On examination, no pulse could be felt at the wrist. The patient, being considered rheumatic, was electrified; and it was only by the obvious heaving of the chain, as it accidentally lay on the breast, that the disease was first suspected, and instantly discovered; the tumour below the clavicle was found about the size of a goose's egg. Near a month intervened before the operation, though determined on, was undertaken, for many obvious and important reasons. Here also we shall pass over the description of the operation, which is minutely, and, we doubt not, accurately detailed. The patient lived about four days, and the following is the account of the appearances on dissection.

"Suppuration was established under the integuments on the fore-part of the throat, anteriorly to the sterno-mastoid muscles. On the thorax being laid open, we discovered an appearance as of suppuration commencing on some masses of fat lying in the anterior mediastinum. The upper lobe of the right lungs was attached to the ribs by close and old adhesions. Between three and four ounces of water were found in the pericardium. The jugular veins, both the external and internal, were of a deeper colour than usual. Heart felt rather soft; right auricle full; right ventricle less full than usual. No disease in any of the valves on either side of the heart. The internal surface of the right auricle, especially on the septum of the auricle, more red than usual. This redness, though of a brighter tinge than that of the jugular veins, was not such as to indicate the existence of inflammation of this cavity. The venous blood was more fluid than usual. Coagula were found in the right and left ventricles, but not uncommon either in their size or other properties.

"On endeavouring to separate the aneurismal sac from the walls of the thorax, it was found to have connected itself so intimately with the second rib, as to have its cavity laid open by an attempt to disunite them. The form of that portion of the bone upon which the aneurism rested, was changed from a flat surface to a cupped form. The extent of the altered part of the second rib was about half an inch.

"The aorta, with the great arteries springing from its arch, and the humeral artery, being removed from the body, were examined next day.

"The aneurismal tumour had extended more in length than in breadth, and had ascended so high as to have reached near to the spot on which the ligature had been applied. The aneurismal tumour was nearly as large as a pullet's egg.

"The artery immediately above the ligature was seen thrown into folds and furrows, which would seem to be the inevitable effect of tying an artery of so great a size. On removing the ligature, and slitting up the artery, its internal coat was found ruptured by the ligature only at one part, to the extent of about one-fourth of its circumference. Through the remainder of the circle, the internal surface of the artery presented a white line, obviously produced by the tightening of the ligature. Two very small portions of coagulable lymph were found immediately above the ligature. The trunk of the artery below the ligature was rather small. The branches of the subclavian artery were not at all enlarged."

Some very judicious remarks follow, which, for the reasons before mentioned, we shall not transcribe. We cannot, however, omit the paragraph with which Dr. Colles closes his paper.

"When an aneurism of the axillary artery shall require this operation, we may indulge a confident hope that the rest of the arterial system is free from disease, as it appears to have been in two of the foregoing cases, that of Levanee, and of the Rev. Mr. S.; one, where the disease arose spontaneously,—the other, where it could be traced to accidental injury. Although this operation has not yet proved ultimately successful, yet I think we should not despair. The history of surgery furnishes parallel instances of operations, now generally adopted, which, in the few first trials, failed of success.

### Art. II.—*Observations on the Fœtal Liver.* By James Bryce, F. R. S. E. &c.

Here are nearly ten pages to show how cleverly it is managed that the fœtal liver shall be large. Even in Haller we are often weary of the politeness with which he compliments a first cause in managing so well for us. Nor can we easily forget how, in this determination to praise every thing, the Baron went a little too far. "The testicles," says he, "remain in the abdomen till after birth, lest such important organs should be injured in the passage of the child into the world." It must either be a strange sort of presentation, or very clumsy management in the midwife, that should render this provision necessary; and accordingly immediately afterwards wards another anatomist shows that they are in the scrotum a month or two before birth. But what most dissatisfies us with this tedious paper is, that the final cause can never be ascertained with any certainty, and it can lead to no practical purpose.

Art. III.—*Reply to the Review of Mr. Baynton's Essay on the Cure of the Crooked Spine in No.* 39 *of this Journal.* By Henry Earle, Surgeon to the Foundling Hospital, &c.

We shall take an opportunity of comparing the original work, and both these reviews, before we offer our opinion on this article.

Art. IV.—*On the Relation subsisting between the Time of the Day and various Functions of the Human Body; and on the Manner in which the Pulsations of the Heart and Arteries are affected by Muscular Exertions.* By Robert Knox, M.D. Edinburgh.

This is a very ingenious and useful paper. As it is unfinished, we shall defer our closer remarks till we see the whole. We hope, however, we may be in time to offer a few hints to the writer. First, we ought to recollect the vast variety we meet with in the human constitution. Respecting the period of the day at which a man may be said to be in his most active condition, (we do not say, "when he has the greatest capabilities," an expression which we hope not to meet with from Dr. Knox hereafter,) some men boast that they never eat their breakfast till they earn it, others complain they can do nothing before that meal; some are fit for business all the morning, others only in the afternoon, and some not till midnight. It is probable that habit may have its influence, as the more fashionable part of the world, particularly females, are often in a state of listlessness till night, when they appear renovated even before they are recruited with stimulating diet of any kind.

Another hint we would offer, relative to an interesting case related in the course of the paper. The body, we are told, continued warm three hours after death, (that is, we suppose, comparatively or sensibly warm to the person who applied his own hand to it,) yet almost every part examined was filled with black blood, and the passage of the blood through the lungs was imperfect. On this we say no more than to offer it to Dr. Knox's speculation; and also, whether he finds the sensible heat of the body, in a state of health, at all influenced by the number of arterial pulsations. These remarks are only proposed to an ingenious writer, that he may use them, or not, as he finds them connected with the object of his experiments, for the result of which we wait with much interest.

Art. V.—*Observations on the Impropriety of returning the Hernial Sac unopened into the Abdomen.* By Henry Edmondston, Surgeon, Newcastle-upon-Tyne.

These observations are not destitute of merit; yet we cannot help expressing a wish that Mr. Edmondston had discovered, before he came to a conclusion, how much he was anticipated by the first Dr. Alexander Monro in the Edinburgh Medical Essays, a work we wish every one to look into for some of the earliest improvements of practical surgery, particularly the papers of that justly celebrated professor.

Art. VI.—*Case of Hydrophobia, which occurred in the Regimental Hospital of his Majesty's 1st Foot, or Royal Scots.* By George Ballinghall, Assistant-Surgeon.

Another melancholy instance of the refractory nature of this disease! Blood-letting had a tolerably fair trial; but, in our opinion, there are two things in which, should a case occur in our practice, we should conduct it differently. First, the patient should be bled in a recumbent posture. In an erect posture, with the sight of the blood, or without it, no one can say how soon even a soldier may faint. In the next place, as mercury was given, if we recollect, in both the supposed successful cases, we cannot reconcile ourselves to its omission. In the same climate, mercury is given for the liver complaint; but it is often necessary to bleed at the same time; and, should that bleeding be carried to any extent, it is never conceived justifiable to omit the mercurial process. All this will be considered only as hints, and not at all to lessen our obligations for the communication, which, though less valuable, has a greater title to our acknowledgments than a cure.

Art. VII.—*Account of Two Trials for Child-Murder.*

These cases, though proper to be recorded, can only be interesting in the neighbourhood in which the events happened, and the legal process followed.

Art. VIII.—*Case of Deafness successfully treated, by the Removal of a Splinter of Wood from the Bottom of the Meatus externus.* By John Stevenson, Esq. Oculist and Aurist to H. R. H. the Princess of Wales, &c.

The case does the writer great credit, and should be carefully perused by every practitioner. The little success usually attending every common mode of treating deafness, is too apt to render us backward in undertaking so unthankful an office. Hence causes are probably often overlooked which diligence and sagacity might discover, and which, when discovered, might be easily removed. What we have said will be a sufficient apology for transcribing the case in Mr. Stevenson's own words.

"Lieutenant-Colonel Webbie Smith, (by whose desire the case is published with his name) of the Royal Horse Artillery, Woolwich, consulted me, on the 25th of last July, respecting a troublesome discharge from his right ear, accompanied with an almost total loss of hearing.

"The following particulars were communicated in reply to my inquiries into the origin of his complaint. A spirited horse, upon which he was riding when on duty near Bayonne, the beginning of May, took fright, and springing from the middle to one side of the road, forced his rider with great violence against some shortened branches of an oak tree, from one of which he received a most severe blow on the right side of his head. The concussion, for a while, so nearly deprived him of his senses, that it was with extreme difficulty he maintained his seat on the saddle. Immediately on recovering himself, he felt a very acute pain in the injured ear. He hastened to camp, and sent for medical assistance. The staff-surgeons found the integuments of the auricle considerably bruised and lacerated, and the concha and meatus externus filled with coagulated blood. The latter was removed by syringing with warm water, and the wound dressed. The local pain and irritation, instead of subsiding, as was expected, rapidly increased, and soon induced a high degree of sympathetic fever, with slight delirium. These symptoms were, in a few days, alleviated, principally by the formation and subsequent escape of a considerable quantity of pus from the meatus internus. After this period, scarcely any uneasiness remained, except on passing the tragus, which was invariably followed by a very pungent and deep seated sensation. This, together with the deafness and discharge, were regarded as the mere consequence of the preceding inflammation; for which the ear was regularly washed with warm milk and water, and various injections, every night and morning. On the arrival of the patient in this country, he took the opinion of several eminent practitioners, and was subsequently recommended to me. An attentive inspection of the organ enabled me to discover something projecting from an accumulation of matter at the farther extremity of the passage, which, by the introduction of a probe, was ascertained to be a solid and slightly moveable substance. The Colonel instantly suggested the probability of its being a portion of fractured and detached bone;—a suspicion he had constantly hinted to his different medical attendants. It was extracted with some difficulty; and, when cleared from adhering discharge, it proved to be a rough angular flat splinter of oak, five lines in length, and three in breadth; one extremity of which, being pointed, had penetrated anteriorly, and in an oblique direction, to the depth of nearly two lines between the cuticular lining and the parietes of the bony canal, close to the membrana tympani.

Some very judicious remarks follow, with a reference to

 other other records, which, in the multiplicity of surgical works, are likely to escape all who do not purposely search for them.

*(To be continued.)*

---

*Anatomie et Physiologie du Systéme Nerveux en General et du Cerveau en particulier avec des Observations sur la Possibilité de reconnoitre Plusieures Dispositions, Intellectuelles, et Morales de l'Homme, et des Animaux, par la Configuration de la Tete.* Par F. I. Gall et G. Spurzheim.

*Anatomie et Physiologie du Systéme Nerveux en General et Anatomie du Cerveau en Particulier.* 1 vol. Paris, 1812.

*Physiologie et Cerveau en Particulier.* 12me. vol.

So long ago as the year 1805, (see our 14th vol. p. 327) we gave a tolerably distinct view of Dr. Gall's Craniology. At that time, our communication with the continent was uninterrupted, and, though the doctor had published nothing himself, we are pleased to find our statement was correct. In the following year, (vol. 15, p. 201,) we were enabled to offer a diagram of the skull in three different views, to illustrate the doctrine which was attended with something like an official report from the House of Correction and Hospital of Torgau, in Saxony. At that time, we could only transcribe reports, which we were careful to do without any comments. In the year 1809, if our readers will turn to our 21st volume, page 149, they will meet with as much as could at that time be collected of Drs. Gall and Spurzheim's opinions, and of their mode of dissecting the brain. Imperfect as that report was, it derived much consequence from the names affixed to it, and also from the minuteness of many parts of the dissections beyond what we are accustomed to in the London schools, where practical anatomy is taught with so many advantages and studied with so much diligence.

The respect with which the mode of dissection, partly taught and partly revived by Dr. Gall, was treated by the Reporters, fairly entitled the opinions, deduced from or confirmed by them, to some attention. But the unsettled state of the continent prevented that regular communication by which only a subject so new could be well understood. At length, one of his coadjutors is arrived in London, has given public lectures, and published his work in English. The question is now a matter of too much notoriety to be any longer overlooked in a Medical Journal. Dr. S. has even given practical proofs of his craneological science in a public hospital in the metropolis.

It is not our intention to enter into the metaphysical part of of these inquiries. Such questions, though, as far as morality is concerned, they may be said to relate to all sciences, yet it is rather to their application than to the mode of learning or teaching them; and, as in a physical point of view, the structure of parts is always the first consideration, to that we shall principally direct our attention.

It appears, that at a very early period of life, Dr. Gall was accidentally directed to an examination of the external form of the head, as indicative of some peculiar mental qualities. In after-life, being destined for medicine, he examined the structure of the brain, and found that the external appearance of the cranium being modelled by the form of the brain, what appeared to him at first only a coincidence might probably be the effect of an original formation of certain parts destined for certain functions; that in proportion to the size of these parts, these functions would be more or less perfect; and also, that those parts would increase by the enlargement or increased number of vessels in proportion as they were oftener brought into action by the exercise of their particular functions.

Avoiding, as we had determined to do, all metaphysical inquiries, we shall endeavour to express ourselves in physical language in this part of the inquiry which relates to the *innate* qualities of the mind. No one who views man as he sees him in any condition of society will doubt, that, however generally the species may be said to resemble each other in intellect and senses, still there are certain differences between individuals which cannot be entirely imputed to education. Even the external senses differ in different individuals, and to each of these we know that certain parts of the brain are appropriated. Why then, it is asked, should we doubt whether the various qualities of the mind depend on particular parts of the same organ, or rather whether the brain does not consist of as many organs as there are faculties, propensities, or passions? whatever answer we may be disposed to give, we cannot surely be inattentive to the minute inquiries of an ingenious individual directed from early life to this single object.

We would not wish it to be supposed, that we are vindicating a doctrine with us at present much too crude to justify us in forming any certain conclusion. Our only intention is, to induce the medical student not hastily to condemn supposed discoveries because they are new; and, perhaps, to induce some of them to select this branch of anatomy and medicine as their peculiar study. By such a division of labour alone can it be perfected. Let us reflect with shame, if not with indignation, that the few solitary cases preserved by by Dr. Marshal are the first regular register which England can boast of anatomical descriptions of the brain of maniacs by a practical anatomist.

In our volume before alluded to, we gave a sufficiently general account of the manner in which these gentlemen conduct the anatomy of the brain. We shall now offer a few extracts to shew the application of their researches to the moral or intellectual faculties discoverable in the formation and magnitude of the various parts.

"One might have expected that anatomists, from observing the great diversity in the constituent parts of the brain, would have been the first to infer a division and plurality of organs for the different intellectual and moral faculties. But when we see, even at the present day, that Vicq-d' Azyr, after having presented not only a positive but a comparative view of the human brain, by commencing with the simplest structure in the insect, gradually ascending to the compound brain of man, and then as gradually descending from man to the insect, still admits but a single organ of the mind, we are taught by experience how little the simple knowledge of a mechanical structure is capable of enlightening either the physiologist, or the philosopher. It is only by attending to the phenomena of nature, and paying no regard to the pre-conceived opinions of cavilling metaphysicians, that we can expect to arrive at any more just or satisfactory ideas concerning the structure of the brain. Herder, struck with the phenomena of the intelligence manifested in different animals and different individuals, conceived the idea of a plurality in the intellectual organs, and even the hope of being at some period able to discover them, by attentively comparing different brains with their different qualities. Bonnet perceived fibres in the brain which he conceived might have different functions.

"We were employed during many years in accumulating a store of physiological and pathological facts, before we could arrive at any rational conclusion on the nature of the brain and nervous system. But to what advantage could we have turned that collection of facts, if we had not previously supposed an intimate and necessary connection with their material conditions? It was thus, that, prepared by the studies of physiology and pathology, we advanced rapidly in discoveries, to which the scalpel alone would never have conducted us. If we have at length been enabled to establish such views of the anatomy of the brain as shall withstand the progress of time, we are indebted for it, almost wholly, to our physiological and pathological ideas. It is the perfect relation between the intellectual phænomena and the material conditions of their existence, that ensures the durability of our anatomical and physiological doctrine relative to the brain and nervous system.

"It will be surely admitted that there is a great difference between asserting that the discovery of the functions of the brain was made independently of the knowledge of its structure, and that there there exists no immediate and necessary connection between its functions and structure. Could it be said, for instance, that motion and secretion have no connection with the organization of the muscles and the viscera, or that digestion and circulation of the blood have not an inseparable relation with the stomach and heart? Have not the organs of the senses an immediate and necessary connection with the functions to be performed by them? Why then should the functions of the brain be excepted from the general rule, for the reason that its organization and functions are of a totally different order from those of the senses, or those belonging to the organic life?

"Any doctrine respecting the functions of the brain, which could be shewn to be in contradiction to its structure, must be false. When any one shall prove that the brain is composed of glands, as an organ of secretion or excretion, it may then, unconnected with any superior function, be ranked with the other organic viscera. When any one shall shew a central point of union for all the medullary fibres, and shall find a cerebral mass alike in all animals, notwithstanding the diversity in number and quality of their faculties, then will the system which maintains a plurality of organs be satisfactorily refuted. If any one shall demonstrate that in different individuals of the same species, notwithstanding the distinctions in their common faculties, the brain shall present an invariable identity in its constituent parts, we shall then no longer assign a seat to the different organs, by comparing their physical development with the intellectual energy. But if, on the other hand, it is an invariable truth, that animals without intellect are also without brain, being provided only with inferior nervous systems, for the supply of the organic viscera, and that these systems are multiplied according as the vegetative or organic life is more complicated; that any faculty of the animal life, as instinct, propensity, intelligence, can only exist in conjunction with brain; that the constituent parts of the brain are multiplied and varied in the same relation and degree as the faculties; that all these facts concur in shewing the extraordinary energy in any particular faculty invariably to correspond with an extraordinary development in some part of the brain; that the derangement of any faculty is connected with a state of disease in a part of the cerebral mass, in the same way that the privation of any of the senses is produced by the existence of disease in its physical apparatus.—Lastly, if it can be shewn as a constant truth, that the brain is composed of many nervous systems, so distinct from each other, that the diversity of the origins, directions, and terminations of the fibrous fasciculi, of which they are composed, can be made evident to the senses, then it must without question be admitted, that an anatomy of the brain is established, in perfect relation to our doctrine of the functions; and the metaphysician will be no longer justified in wandering abroad in vague speculations, by asserting that the operations of the mind are too obscurely hidden to allow of our discovering the material form which indicate such operations."

This

This is all the notice we shall at present take of this new system of physiology and pathology in an organ, the parts of which have hitherto only been distinguished by whimsical appellations, and the very form of which has in our own time been matter of dispute between learned and enlightened anatomists. In our next, we shall take a view of the *Physiognomical System of Drs. Gall and Spurzheim, founded on an Anatomical and Physiological Examination of the Nervous System in general, and of the Brain in particular, and indicating the Dispositions and Manifestations of the Mind; by J. G. Spurzheim, M.D.*

---

### *An Essay on Dew and Several Appearances connected with it.* By William Charles Wells, M.D. F.R.S.

This valuable series of well imagined and well conducted experiments, with their results, is too important to be overlooked by us. When we consider the necessary daily engagements of the author, his precarious health, and the extreme diligence and fidelity with which the whole has been conducted, we cannot fail to express our gratitude for the acquisition derived from his nightly labours. All the experiments being related with as much brevity as is consistent with perspicuity; we can make no other abstract, than by giving the results and the inductions made by himself, particularly directing the reader's attention to every practical inference which may occur.

The first section is " On Circumstances which Influence the Production of Dew." For the production of dew, the author found the most favourable state of the evening was calmness of the air and clearness of the sky. In windy and cloudy weather, dew is never formed; but a small quantity may be found with wind on a clear night, or with clouds on a calm night. On a clear night, a very light breeze rather facilitates the production of dew. Dew begins to form on grass in shady places soon after the atmosphere is cooled, and continues to be formed a very short time after sun-rise, if the weather is favourable during the whole night. On smooth dense substances, the formation resembles the condensation of an evaporating fluid on similar substances. The quantity of dew, other circumstances being equal, is proportionate to the quantity of moisture with which the atmosphere is loaded; consequently it is for the most part more abundant in westerly and southerly winds, with a favourable state of the atmosphere and sky. From hence the author offers a proposition which, we believe, is not universally admitted by practical meteorologists: namely, that dew is less abundant during a long series of dry weather.

weather. It must, however, be admitted, that this is so rare in England as to render any conclusion very difficult; it must be added, too, that the author does not speak of any increase of dew before, but after, rain. From every aggregate circumstance, however, Dr. W. does not scruple to give it as an aphorism, that, *cæteris paribus*, there is more dew in proportion as the air is more loaded with moisture.

Secondly, Dew is more copious in those nights favourable for its production as above-described, which are followed by misty or foggy mornings.

Thirdly, In nights of the above description dew is more copious in proportion as the atmosphere is hot. This rarely occurs in this country, but is inferred from our knowledge of the capacity of watery vapour to diffuse itself in heated air, and the precipitation which follows the subduction of that heat; and, it is proved, by the observations of all travellers, during the night in warmer climates.

Lastly, All other events remaining the same, more dew was found to be formed between midnight and morning than between evening and midnight, which is imputed to the greater coldness of that part of the 24 hours.

The remainder of this section goes to prove, that the formation of dew was in all cases proportionate to the free exposure of a substance to the aspect of a clear sky, and, in many cases, to the height at which it was elevated above the ground.

The next section is, "Of the Cold connected with the Formation of Dew." On this subject, the author, after a few remarks on the general admission of poets and philosophers that dew is cold, informs us, that in all clear nights, the ground covered by grass is warmer sometimes by more than 12 degrees than the atmosphere a few feet higher, and that this difference will frequently vary according as the sky may be more or less clear from clouds. The turbidness from fog produced so similar a condition on every part, that the thermometer discovered very little difference, whether on the ground or elevated above it. He found also, by the most delicate experiments on wool and other loose fibrous substances, that the comparative quantity of dew collected on each was proportionate to the degree of cold. The increased cold on the surface of the earth was very much confined to spots covered by grass, which was often 10 or 12 degrees colder than gravel walks or bare garden mould. The temperature of the earth, an inch or even half an inch below the surface, was found sometimes from 12 to 16 degrees warmer than the grassy surface. On the top of Dr. Wells's town house, he found wool lying on a wooden frame colder than the surrounding air, and that it had imbided dew. Metals also furnish another proof of the connection of dew with cold.

The remainder of this section is devoted to an examination into the capacity of different substances to acquire a temperature colder than the surrounding atmosphere.

Dr. Wells found the grass, however evenly shorn, too uncertain for his accurate inquiries. He found too that all filamentous substances cooled faster, and to a greater degree, than any others. Of all others, swan's down remaining on the skin of the bird was the most steady, and cooled the most, being in one experiment 20° below the temperature of the atmosphere above it, which is four degrees more than the most accurate philosophers have ever discovered in snow. In this and in all other experiments, it was found that the slightest opake covering of the substance considerably lessened the degree of cold.

The second part is "On the Theory of Dew." Aristotle's opinion, that dew is a species of rain formed in the lower atmosphere, has been sanctioned by no less a name than Mr. Leslie's, of Edinburgh. Dr. Wells, however, proves its fallacy, by shewing, that when bodies on the ground remain dry, others elevated a few feet will be covered with dew. Metals remaining free from dew whilst other bodies are covered with it, gave rise to an opinion, that dew was formed by electricity. Dr. W. though he admits that every change in a body is probably attended with some electric effect, yet shews, by a variety of unanswerable objections, the fallacy of the above opinions. All the other arguments, it is observed, overlook one important fact, namely, the connection of cold with the production of dew. Former writers have considered this coldness as the effect, but Dr. W. clearly proves, that when other circumstances are equal, every substance will be dewed in proportion as it is previously cooled. Some very accurate observations follow, all shewing that substances imbibing dew, or retaining it on their surface, are cooled before they receive dew, and sometimes are warmer afterwards. We cannot pursue our author's reasoning throughout, but may be allowed to remark in general, that, on the earth, the increase of cold is in proportion as the sky is more clear. That the cause of dew is the precipitation from the atmosphere of water, which it retained when more heated, and not merely the effect of a continued evaporation from the earth. Hence, that the vulgar language is, in this as in many other instances, more correct than the language of philosophy, and that dew actually falls instead of rising.

Having

Having thus shewn that bodies attract dew in proportion as they are previously cooled, the next enquiry is, why grass should attract more dew than the bare earth? or, why substances elevated a little above the grass should be colder than the grass itself? To account for this, Dr. Wells has recourse to the discoveries of Mr. Leslie and Count Rumford; by the first we learn satisfactorily, that bodies cooled or heated radiate their cold or heat; and, by the latter, that air is a bad conductor of heat. If, therefore, during the summer, the upper regions of the atmosphere are, as we now know to be the case, colder than the earth, when there is no impediment, that is, when there are no solar rays, no uncertainty from disturbed elements, no interruption to the transmission of heat or cold by radiation of either from the earth or the sky; the former will be constantly radiating its heat upwards, and the latter its cold downwards. But, air being a bad conductor of heat, whatever portion the earth may radiate, will very slowly pass upwards, even to the higher fibres or blades of the grass; hence the immediate surface of the ground will remain warmest, and the greatest quantity of dew will be precipitated on the highest surface of the grass. This difference will be still more striking in a substance not at all, or very slightly, attached to the earth, and elevated a few feet; hence fibrous substances, suspended in the air, will receive a full quantity of radiated cold from the sky, and little or none of the radiated heat from the earth.

Such is the grand outline of this valuable little performance. In this manner, too, the author accounts for the formation of ice in the East Indies, not by the cold produced from evaporation, but by the cold radiated from the sky; by the care too that is taken to prevent the communication of heat radiated from the earth, and by the necessity that is found of a tranquil state of the atmosphere for the success of the process. In this manner, also, is accounted for the non-appearance of dew on any naked part of the human body. The capacity of maintaining its own standard heat prevents that previous cooling which is necessary for the condensation of the precipitated moisture of which dew consists.

From all these facts, we may draw some important medical inferences; that a substance, however thin, interposed between the sky and the earth, may prevent the radiation of heat, or of cold, from or to the body thus covered, and, consequently, the formation of dew by a reduced temperature; hence the advantage of a canvas tent, and a covering,

 however, however slight, to protect plants, and to prevent the congelation of water in leaden pipes.

There are some arguments to prove, that the sunbeams may heat the atmosphere in any degree of purity; we conceive this can scarcely be doubted, as far as is necessary for our author's reasoning, for whether the atmosphere is heated, or whether heat is diffused in it, is sufficient for all that is required by him. Now we conceive, that, in the calmest weather, the sun daily darting on the Atlantic Ocean, in a calm state, must suffer a reflection of its calorific rays, which, in passing upwards, must, in a certain degree, affect the atmosphere; or the surface of the water reflecting the heat, as well as light, will produce a sensible effect on every opake substance on which it strikes. When we offer this conjecture, we do it, subject to the correction of one, who has reduced every thing as far as it can be so reduced to actual experiment, and who, as far as we can judge, has overlooked nothing which may tend to the perfection of his ingenious theory.

---

*Observations on the principal Diseases of the Rectum and Anus, particularly Stricture of the Rectum, the Hæmorrhoidal Excrescence, and Fistula in Ano.* By Thomas Copeland, Fellow of the Royal College of Surgeons, and Assistant-Surgeon to the Westminster General Dispensary. Second edition, considerably enlarged. 8vo. pp. 183. Callow, 1814.

The favourable opinion which we gave of the first edition of this work is confirmed by a second edition being so soon demanded. We have now, therefore, only to notice the new matter contained in the present volume, which increasing practice has enabled the author to supply. The first addition that we observe, is the section which treats "on the consequences produced by the irregular or too powerful action of the sphincter muscles." This is a very frequent affection, yet the author remarks, and we know his reading is extensive, that he is not aware that it has been described by any medical or surgical writer. This, perhaps, may be explained by the fact, that physicians and surgeons formerly were less at a loss for subjects to write upon, than in the present age, when a trifling case, or some unexpected success from a remedy, once in use, then discontinued, and again employed, forms the subject of a considerable treatise. Some men, too, of extensive experience never write, and what they have observed during a long course of practice, unfortunately dies with them. We believe also that, though the the affection is frequent, it seldom occurs in the severe degree in which Mr. Copeland has described it; and from its being known that he has devoted much attention to diseases of the rectum, it is likely that such cases may occur more frequently to him than to other practitioners. After commenting upon the different degrees of strength, and the variety of structure of the sphincter in individuals, and observing that those in whom the muscle is strong, are predisposed to diseases of this part, he proceeds,

"When the sphincter muscle of this powerful and extensive kind is excited, by the efforts to pass the stools, to a high degree of inflammation, perhaps there is no disease that the human frame is subject to which is more painful; its involuntary contractions are compared to the pains of labour; they frequently come on immediately, but more usually about an hour or more, after each evacuation, and sometimes continue till the succeeding one: in some instances, the complaint goes on to produce suppuration, and consequent fistula; sometimes the irritation is propagated to the neck of the bladder, and produces a retention or impediment to the urine. I have seen it, in two cases, extend up the canal, and give rise to attacks of violent cholic, and an increased secretion from the whole inner membrane of the gut, so that an evacuation of mucous cylinders, of the size of the part of the canal where they are formed, or of detached pieces of mucus, are seen mixed with the fæces; yet all this has been finally removed by the bougie. I have seen it followed by a constant evacuation of shreds of coagulable lymph, which has continued through life, and produced the greatest distress. When this substance accumulated in the bowels, it was accompanied with pain, which continued till it was discharged."

The treatment recommended by Mr. Copeland is the use of the bougie, opium, and occasional purgatives. It is a large bougie, however, on which he chiefly relies. We need hardly remark, that the introduction of a candle up the rectum is of ancient and vulgar use for obstinate constipation and contraction of the sphincter muscle. Several cases are related by Mr. C.: the following is the first, and the only one which our limits will permit us to copy.

"I was consulted by a barrister of eminence for a complaint of the rectum, which he had been afflicted with for many years. He was about forty years old, and otherwise in good health; but so slow and imperfect was the action of his bowels, that he could never pass his fæces without the assistance of a purgative medicine, and very long-continued efforts. These efforts frequently excited pain and inflammation, which disqualified him for his business for many days; but, on common occasions, a considerable portion of his time was wasted in efforts to evacuate the bowels. Sometimes he passed well-figured stools; at others, they were small, or liquid. There

There was no discharge, or protrusion of the membrane from the anus. A bougie passed with difficulty through the sphincter muscle, till it encountered the os sacrum, projecting into the pelvis; and, after some time, went on in the canal of the gut.

"On examination, the finger was compressed with great violence by the sphincter muscle, for more than two inches; that is, above twice as much as its usual breadth. I told him, that I did not believe there was any serious stricture of the rectum; but that he would obtain relief by using very large bougies only through the sphincter muscle; at least, that it was advisable to commence with this simple treatment. The introduction of the bougie gave him great pain at first, but became every time easier, and he soon experienced greater facility in passing his stools; so much, that, in two months, he was able almost to do without purgative medicines, and but little effort was required in evacuating the bowels."

A new section, containing some good practical remarks on Prolapsus Ani, is introduced; and the author has also added a section on Ulceration of the Mucous Membrane.

"The inner membrane of the rectum (he observes) is often affected with ulceration, which is more or less important, in proportion as it is extensive, and as it is situated farther from or nearer to the anus. The principal characters and symptoms of the disease also are derived rather from the part of the gut that is affected, than any peculiarity of the affection. If it is situated quite above the sphincter muscle, it excites but little pain or inconvenience, although it may have advanced to a considerable size.

"A quantity of purulent matter is passed before each stool, and sometimes without any fæces accompanying it. For a very considerable time, the influence it has on the general health or comfort of life is not very striking; indeed, the disease, on this account, is the more alarming, as it has often arrived to a serious degree before it attracts notice from the pain or inconvenience it produces. When, on the contrary, the sphincter muscle is involved in the disease, there are, either from the first attack, or after a short period, very frequent and very painful tenesmus, and incontinence of stool, which keep the patient out of society, and destroy all his enjoyments. If it is confined to the mere surface of the membrane round the anus, there is usually an exudation of purulent fluid through the anus. It is often attended with, and, perhaps, produces a tendency to, prolapsus ani, from the irritation and straining it excites, and the protruded part has a bright red and abraded aspect."

"The appearance which the disease exhibits in dissection is various. Sometimes there is one large ulcerated cavity; in other cases, the rectum is studded with spots of ulceration of various sizes; but those cases which have been produced by a severe attack of dysentery, have often an extensive surface of the canal in a state of ulceration. Sometimes it has a more mild character, and so so superficial as to be visible, and within the reach of applications which will relieve it."

"In the treatment of this complaint, our means of cure are more certain and more effectual, as the disease is nearer the anus; but the necessity there is of daily performing the natural functions of the part, renders it always a disease of difficult cure. Richter has very strongly recommended the injection of a decoction of hæmatoxylum, which is sometimes used with benefit. Mercury, in small and long-continued doses, has, in some instances, been beneficial; and various kinds of mercurial applications, such as aq. calcis cum hydr. submur. or a weak solution of hydrarg. muriat. used as an injection. The expressed juice of the carrot is very generally recommended; but, I believe, more benefit is expected from it than it is found to produce. When the ulcerations approach towards the external surface, I have seen them often heal under the use of equal parts of aq. lytharg. acet. et ol. oliv. when they had resisted every other remedy for a considerable time. But the ulceration of the more interior parts of the canal is always a serious disease, often baffling all our efforts to relieve it."

Besides the new matter already noticed, we observe several additional cases of the affections treated of in this volume; and we have no hesitation in saying, that it constitutes an important accession to our knowledge of a very obscure class of diseases.

---

*The Institutions of Physiology, translated from the Latin of Professor Blumenbach, with additional Notes, illustrative and emendatory.* 8vo. pp. 260. Cox and Son. Lond. 1815.

The Institutions of Physiology, by Blumenbach, is a work well known, and has long since obtained the meed of public approbation. The original had become scarce in this country, and a translation was much wanting. The leading characteristics of the work are accuracy and conciseness; if it does not abound in curious speculations and brilliant conceptions, it is unincumbered with obsolete notions and vague conjectures. The author, indeed, has chosen to introduce a section upon the mental faculties, which might as well have been omitted, for he comprises all his information on this subject within the compass of two pages. The meanest dictionary would give the student more knowledge of the mental phenomena than he can find in the work before us. Let us, for example, adduce his account of reason, which he places last in the scale. "Lastly, reason, the most noble and excellent of all the faculties, draws inferences from the comparisons of the judgment." Bailey, in his dictionary (ed. 1751), expresses the same notion in fewer words, and in a happier manner, manner, viz. "that faculty of the soul whereby we judge of things."

In general, the translator has done justice to the author, but in some passages he has shewn negligence, and in one instance has perverted the meaning intended, by rendering *animantibus* animals, instead of beings endowed with life. We would also reprove his frequent omission of the author's references, which are rich, and might open to the student many curious volumes. To make amends, he has added some valuable notes, which are the more necessary, as several discoveries have been made since Blumenbach wrote. As a specimen of these, we shall extract the note at the end of the section on animal heat.

"No phenomenon in living bodies is more remarkable than their peculiar temperature, and no one was of more difficult solution before the progress of modern chemistry. If two different bodies are placed in a temperature higher or lower than their own, for a certain length of time, they will, at the end of the period, be found not of the same, but of different, temperatures. That which has the higher temperature, is said to have a smaller capacity for caloric; that which has the lower, a greater capacity. To raise the former to a given temperature, therefore, requires less heat than to raise the latter to the same degree.

"The temperature of solids is more easily affected by a given quantity of heat, than that of fluids: or, in other words, solids have a smaller capacity for caloric than fluids, and fluids than aeriform bodies. If, therefore, a solid becomes fluid, or a fluid aeriform, it absorbs a great quantity of heat, though its temperature remain precisely the same. And the converse holds equally good, if an aeriform substance becomes liquid, or a liquid solid, the heat which it before contained is now (from the diminished capacity of the body) much more than sufficient for the temperature which before existed, and the temperature of the body accordingly rises.

"In respiration, the dark blood of the pulmonary artery parts with a portion of its carbon, and acquires a florid hue. This carbon unites with the oxygen of the inspired air, and forms carbonic acid, which is expired with the other constituent of the atmosphere (nitrogen or azote), which appears to have experienced no change from inspiration.

"Dr. Crawford rendered it probable, by his experiments, that the arterial blood has a larger capacity for caloric than the venous; and common air, than carbonic acid gas. When, therefore, the carbon of the venous blood unites with the oxygen of the air, and forms carbonic acid, the less capacity of this than common air for caloric, must cause an increase of temperature, but the blood having changed from venous to arterial, has acquired a greater capacity than before, and absorbs the heat given out by the carbonic bonic acid. The blood, of course, does not become warmer, because the heat is not more than sufficient to render its temperature equal to what it was previously; and indeed it is not quite sufficient for this, for the arterial blood of the pulmonary veins is generally two degrees lower than that of the pulmonary artery.

"The body in this way acquires a fund of heat, and yet the lungs, in which it is acquired, do not experience any elevation of temperature.

"The arterial blood, charged with much heat which is not sensible, circulating through the small vessels, becomes venous, acquires a dark hue, and its capacity for heat is diminished; consequently its temperature rises: the heat which was previously latent, is, from the decrease of capacity, sufficient to raise its temperature, and is evolved. In this mode, the loss of heat which occurs from the inferior temperature in which we live, is compensated. The fresh supply is taken in at the lungs, and brought into use in the minute vessels.

"Of late, this theory has been brought into discredit, by the experiments of M. M. De la Roche and Berard, on the capacities of bodies for caloric, and by those of Mr. Brodie upon respiration. All experiments upon the capacities of bodies by heat, are very delicate, and liable to error; and the conclusions of Crawford on this point, with respect to the gases, have been disproved by the French chemists.

"Mr. Brodie cut off the communication between the brain and lungs of animals, and continued respiration artificially.* The usual chemical changes continued in the lungs upon the blood, nevertheless the temperature of the animals diminished, and even more rapidly than if the respiration had not been continued, owing, he says, to the succession of cool air sent into the lungs. He, therefore, concludes, that animal heat depends much more upon the nervous energy than upon the chemical changes of the blood. But many circumstances favour the doctrine of Crawford. In high temperatures we have less necessity for the evolution of heat, by the chemical changes of the blood and air; whereas, in low temperatures, as more heat is required, to sustain the natural degree of temperature, the chemical changes are more necessary; accordingly, in very high temperatures, the arterial blood remains arterial, is as florid in the veins as in the arteries, and the inspired air is less vitiated; in low temperatures the venous blood is extremely dark, and the inspired air more vitiated. Vide Crawford on Animal Heat, p. 387, et seq.

"Dr. Crawford states, that the chemical process of respiration may, in certain cases, be the means of cooling the body. If the pulmonary exhalation is in very great abundance, it will carry off so much of the heat given out during the change of the oxygen into carbonic acid, that there may not be sufficient to saturate the

---

"Philos. Trans. 1812."

increased capacity of the arterial blood; this will, therefore, absorb heat from the system, as it passes along, till its temperature equals that of the other parts. Animal Heat, p. 388.—Now, in Mr. Brodie's experiments, the influence of the brain was cut off from the lungs; these must have been weakened, and, as a most ingenious friend of the translator's remarked, the mouths of the relaxed vessels would consequently exhale vapour in great abundance, and carry off much of the heat which ought to have assisted to saturate the increased capacity of the arterial blood; this must have acquired heat from the interior of the system, and the general temperature of the body of course fell. In the animals whose respiration was not kept up artificially, what fluid was secreted must have remained in the liquid form,* and, not being carried off by expiration, could not produce that diminution of temperature which was observed in the instances where respiration was artificially sustained.

"It may not be improper to remark here, that Dr. Prout wrote a paper last year, in Dr. Thomson's Annals of Chemistry; and Dr. Fyfe published, about the same time, an inaugural Dissertation at Edinburgh, upon the variety of the quantity of carbonic acid gas formed in the lungs under different circumstances. The former found the quantity to increase from day-break till noon, and then gradually diminish again till morning. The increase took place exactly at day break. Whenever the quantity had been diminished, it afterwards rose proportionally, and *vice versâ.* It was diminished by whatever contained alcohol, by tea, depressing passions, fatigue, mercury, and probably during sleep.

"An extensive set of experiments are necessary on this point, made at different ages, in different temperaments, climates, temperatures, and under every variety of circumstances.

Here we had proposed to close our account of a celebrated and well-known work, but we cannot forbear pointing out an absurdity in the author, which his good sense ought to have enabled him to avoid. We allude to the subject of generation, a subject which physiologists, in common with young people of either sex, seem peculiarly fond of; in fact, it occupies the longest section in the present work, yet the most acute physiologist cannot exceed the common facts detailed by anatomy, unless he descends to conjecture. Blumenbach asserts,

"528. Semen has also this peculiarity, first observed by L. Ham Dantiscan, in the year 1677, of being animated by an infinite num-

---

"* Dr. Le Gallois (Experiences sur le principe de la vie, 246) actually found this invariably the case. 'The lungs,' he says, 'are always found swollen, and filled with a frothy fluid,' after decapitation, or the division of the eighth pair. In his experiments, respiration was not supported artificially."

ber ber of small worms visible by the microscope, of the kind denominated infusoria, and of different figures in different kinds of animals. In man, these spermatic animalcules are oval, and have very fine tails. They are said to be found in prolific semen only, so that they are, in some degree, an adventitious criterion of its prolific maturity. I say adventitious, because I hope there is no necessity, after so many weighty arguments and observations, at present, to remark, that they are not the fecundating principle, and much less the germs of the future offspring."

Now, if the author does not know this to be absolutely false, he is unfit for a teacher of physiology. If the semen was *animated* with small worms, of course they must be living beings, but where and how are they generated? We conceive the act of secretion, so far from producing animalculæ, would be destructive to them, and have some doubts whether worms could exist in the slime of human semen; at all events, we would be glad to know what becomes of these worms in copulation, on the principle that nothing is created in vain. The remains of man may ultimately nourish worms, but we have yet to learn that worms contribute to the formation of man. The notion of animalculæ in semen, however, is not peculiar to Blumenbach; most foreign physiologists will support his authority. Richerand, and we should suppose a Frenchman would be tolerably correct on such subjects, observes, "Examinée au microscope, la semence offre de petits animalcules, ayant une tête arrondie, une queue effilée, et se mouvant avec célérité. Est ce à leurs mouvemens que seroit due la liquéfaction des parties gluantes et filamenteuses du sperme? Ces animalcules microscopiques ne se voient dans le liquide séminal, qu'à l'epoque de la puberté. On a cru remarquer qu'ils fuyoient la lumière, et decrit leurs mœurs, leurs habitudes, et même leurs maladies." Thus one writer copies from another, and error is propagated.

Considering the advanced state of chemistry and anatomy in this country, it is time that we had a standard work on Physiology of British manufacture.

